# A New Therapeutic Assessment Score for Advanced Hepatocellular Carcinoma Patients Receiving Hepatic Arterial Infusion Chemotherapy

**DOI:** 10.1371/journal.pone.0126649

**Published:** 2015-05-20

**Authors:** Issei Saeki, Takahiro Yamasaki, Norikazu Tanabe, Takuya Iwamoto, Toshihiko Matsumoto, Yohei Urata, Isao Hidaka, Tsuyoshi Ishikawa, Taro Takami, Naoki Yamamoto, Koichi Uchida, Shuji Terai, Isao Sakaida

**Affiliations:** 1 Department of Gastroenterology and Hepatology, Yamaguchi University Graduate School of Medicine, Ube, Yamaguchi, Japan; 2 Department of Oncology and Laboratory Medicine, Yamaguchi University Graduate School of Medicine, Ube, Yamaguchi, Japan; 3 Department of Internal Medicine, Nagato General Hospital, Nagato, Yamaguchi, Japan; 4 Department of Human Nutrition, Yamaguchi Prefectural University Faculty of Nursing and Human Nutrition, Yamaguchi, Yamaguchi, Japan; Yonsei University College of Medicine, KOREA, REPUBLIC OF

## Abstract

**Background & Aims:**

Hepatic arterial infusion chemotherapy (HAIC) is an option for treating advanced hepatocellular carcinoma (HCC). Because of the poor prognosis in HAIC non-responders, it is important to identify patients who may benefit from continuous HAIC treatment; however, there are currently no therapeutic assessment scores for this identification. Therefore, we aimed to establish a new therapeutic assessment score for such patients.

**Methods:**

We retrospectively analyzed 90 advanced HCC patients with elevated baseline alpha-fetoprotein (AFP) and/or des-gamma-carboxy prothrombin (DCP) levels and analyzed various parameters for their possible use as predictors of response and survival. AFP and DCP responses were assessed after half a course of HAIC (2 weeks); a positive-response was defined as a reduction of ≥ 20% from baseline.

**Results:**

Multivariate analysis identified DCP response (odds ratio 16.03, *p* < 0.001) as an independent predictor of treatment response. In multivariate analysis, Child-Pugh class A (hazard ratio [HR] 1.99, *p* = 0.018), AFP response (HR 2.17, *p* = 0.007), and DCP response (HR 1.90, *p* = 0.030) were independent prognostic predictors. We developed an Assessment for Continuous Treatment with HAIC (ACTH) score, including the above 3 factors, which ranged from 0 to 3. Patients stratified into two groups according to this score showed significantly different prognoses (≤1 vs. ≥2 points: median survival time, 15.1 vs. 8.7 months; *p* = 0.003).

**Conclusions:**

The ACTH score may be useful in the therapeutic assessment of HCC patients receiving HAIC.

## Introduction

Hepatocellular carcinoma (HCC) is the fifth most common cancer and the second leading cause of cancer-related deaths worldwide [1]. Recent advances in treatment techniques, including hepatic resection, percutaneous ethanol injection (PEI), radiofrequency ablation (RFA), transcatheter arterial chemoembolization (TACE), hepatic arterial infusion chemotherapy (HAIC), sorafenib administration, and transplantation, have improved the prognosis of this malignancy [2–7]. However, the prognosis of advanced HCC patients, especially in the presence of vascular invasion and/or extrahepatic spread, remains poor.

For patients with advanced HCC, the multikinase inhibitor sorafenib is recommended as the current standard of care [8]. In contrast, HAIC is one of the recommended treatments in Japan [9]. However, there are no established criteria for the selection of HAIC or sorafenib in the treatment of advanced HCC. In addition, no randomized controlled trials have been conducted to compare these treatments. Although sorafenib has been shown to improve survival in advanced HCC patients with preserved liver function, the response rate of sorafenib therapy is low, at approximately 2–3% [7, 10]. In contrast, the response rate for HAIC therapy is approximately 30–40%, and survival is significantly longer in HAIC responders than in HAIC non-responders [11–13]. The median survival time (MST) is longer in patients who undergo HAIC using low-dose FP (cisplatin and 5-fluorouracil; 14.0 months) than in patients who do not receive active therapy (5.2 months; *p* < 0.0001) [14]. Previously, our retrospective study of HAIC demonstrated a response rate of 36% and an MST of 10.2 months, with significantly longer survival in responders (MST in responders, 17.8 months; non-responders, 7.2 months; *p* < 0.0001) [15]. Because of the poor prognosis of HAIC non-responders, it is important to identify patients who may benefit from continuous HAIC treatment. Although staging systems for HCC can predict patient prognosis [16, 17], there are currently no therapeutic assessment scores to aid decision-making with regard to continuous HAIC treatment. Therefore, we aimed to establish a new therapeutic assessment score for such patients.

## Patients and Methods

### Patients

Between July 1997 and July 2012, HAIC based on low-dose FP was administered to 130 patients admitted to our hospital with unresectable HCC. HCC was considered unresectable in cases of bilobar disease, extrahepatic metastasis, portal vein tumor thrombosis (PVTT), or locally advanced disease that was too extensive for resection. A diagnosis of HCC was based on imaging results and on elevated serum levels of alpha-fetoprotein (AFP) and/or des-gamma-carboxy prothrombin (DCP). Of the 130 patients, 90 patients with elevated baseline levels of AFP (≥ 20 ng/mL) and/or elevated baseline levels of DCP (≥ 40 mAU/mL) were enrolled in this retrospective cohort study. This study (H24-31) was approved by the Institutional Review Board of Yamaguchi University Hospital, and written informed consent was obtained from all patients before inclusion in the study. The study protocol was conducted according to the principles of the 1975 Declaration of Helsinki.

### Tumor Stage and PVTT Grading

Tumor staging (T factor) was performed according to the Liver Cancer Study Group of Japan criteria, and based on whether the tumor was (1) solitary, (2) no greater than 2 cm in diameter, and (3) without vascular invasion. Stage I was defined as fulfilling all three conditions (T1), stage II as fulfilling two of the three conditions (T2), stage III as fulfilling one of the three conditions (T3), stage IV-A as fulfilling none of the three conditions (T4) with no distant metastasis or any T factor with lymph node metastasis, and stage IV-B as any T factor with distant metastases.

PVTT grading and hepatic vein tumor invasion grading were also assessed according to the criteria of the Liver Cancer Study Group of Japan [18, 19]. PVTT grading was based on the location of the tumor thrombus in the peripheral portal vein: Vp1, tumor thrombus in a third or more of the peripheral branches of the portal vein; Vp2, tumor thrombus in a second branch of the portal vein; Vp3, tumor thrombus in the first branch of the portal vein; or Vp4, tumor thrombus in the trunk of the portal vein. Hepatic vein tumor invasion grading was based on the location of the tumor thrombus in the hepatic vein: Vv1, tumor thrombus in any peripheral branch of the hepatic vein; Vv2, tumor thrombus in the posterior inferior hepatic vein trunk, short hepatic vein, or right, middle, or left hepatic vein trunk; or Vv3, tumor thrombus in the inferior vena cava.

### Catheter Placement

A heparin-coated 5-French catheter (Anthron P-U Catheter; Toray Medical Co. Ltd., Tokyo, Japan), connected to a subcutaneously implanted reservoir, was inserted intraluminally from the femoral, subclavian, or brachial artery and was positioned in the proper or common hepatic artery. The gastroduodenal and right gastric arteries were occluded with steel coils to prevent gastroduodenal injury from the anticancer agents. The entire procedure was performed under local anesthesia. The reservoir device was filled with 5 mL (5000 units) of heparin solution every 2 weeks to prevent arterial occlusion.

### Chemotherapeutic Regimen

The patients received repeated arterial infusion of chemotherapeutic agents via the injection port. Patients were treated with either HAIC using low-dose FP [20, 21], HAIC using low-dose FP with isovorin [22], HAIC combination therapy consisting of low-dose FP with isovorin and subcutaneous interferon (IFN)-alpha 2b [23], or HAIC combination therapy consisting of low-dose FP with isovorin and subcutaneous pegylated-interferon (PEG-IFN)-alpha-2b [24]. We previously reported no significant differences in response and survival between these four regimens [15].

One course of chemotherapy consisted of 5 consecutive days (days 1–5) of daily cisplatin administration (10 mg/body; Nippon Kayaku Co., Tokyo, Japan), followed by 5-fluorouracil (250 mg/body; Kyowa Hakko Co., Tokyo, Japan). Isovorin (6.25 mg/body; Wyeth KK, Tokyo, Japan or Kyowa, Hakko Kirin Co., Tokyo Japan) was administered daily on days 1–5. IFN-alpha-2b (3 MU/day; Intron A, Schering-Plough KK, Osaka, Japan) was administered on days 1, 3, and 5, and PEG-IFN-alpha-2b (50 μg; PegIntron, Schering-Plough KK) was administered on day 1. Days 6 and 7 were rest days. This course was repeated for 2 weeks, suspended for 1 week, and then repeated again for 2 weeks. In one treatment cycle, arterial infusion chemotherapy was administered 20 times, IFN-alpha-2b was administered 12 times, and PEG-IFN-alpha-2b was administered 4 times. Both cisplatin and 5-fluorouracil were administered using a mechanical infusion pump set at 1 h and 5 h, respectively. Isovorin was administered at 10 min. The serotonin antagonist ondansetron hydrochloride (4 mg; Zofran, GlaxoSmithKline, Tokyo, Japan) was administered intravenously as an antiemetic agent.

### Evaluation of Treatment Response

The evaluation of the response to treatment was classified according to the RECIST guidelines ver.1.1 [25]. Dynamic computed tomography or magnetic resonance imaging was performed before and after one course of HAIC. Complete response (CR) was defined as the disappearance of all target lesions. Partial response (PR) was defined as a decrease of at least 30% in the sum of the longest diameter of the target lesions compared to the pretreatment reference value. Progressive disease (PD) was defined as an increase of at least 20% in the sum of the longest diameter of the target lesions. Treatment response was classified as stable disease (SD) if none of these criteria were met. Patients who had not completed the first cycle of therapy were regarded as having PD if radiological disease progression was confirmed at that time.

AFP and DCP serum levels were measured using the LiBASys automated immunologic analyzer (Wako Pure Chemical Industries Ltd., Osaka, Japan). Both were obtained at baseline (prior to the administration of HAIC), 2 weeks after initiation of HAIC (half course of HAIC), and at the end of one course of HAIC (full course of HAIC). An AFP positive-response was defined as a reduction in serum AFP of more than 20% from baseline after half a course of HAIC [26, 27]. Similarly, a DCP positive-response was defined as a reduction in serum DCP of more than 20% from baseline after half a course of HAIC. A non-response was defined as a reduction of < 20% from baseline and an increase (into the abnormal range) from the baseline of AFP or DCP level after half a course of HAIC.

### Statistical Analysis

The data are expressed as the mean ± standard deviation. Univariate and multivariate analyses of the predictors of response were assessed by logistic regression analysis. We assessed 13 variables in the response, including gender (male or female), age (younger or older than 65 years), the presence of anti-hepatitis C virus antibody, hepatic reserve capacity (Child-Pugh score A or B), extrahepatic metastasis (yes or no), tumor stage (stage II-III or IV), previous treatments (yes or no), PVTT grade (Vp 0–2 or Vp3-4), inferior vena cava invasion (yes or no), AFP level (< 1000 or ≥ 1000 ng/mL), DCP level (< 1000 or ≥ 1000 mAU/mL), AFP response (yes or no), and DCP response (yes or no). Overall survival was calculated using the Kaplan–Meier method. The clinical data were assessed as predictors of survival using univariate and multivariate Cox proportional hazard regression analysis. Survival time was defined as the interval between the first HAIC and the last follow-up or death. The follow-up period ended on December 31, 2013. Statistical significance was defined as a *p*-value < 0.05. All analyses were performed using the JMP ver. 10.0 software package (SAS Institute, Cary, NC, USA).

## Results

### Patient Characteristics

The clinical profiles of the 90 HCC patients treated with HAIC are summarized in Table 1 and S1 Table. The patient population consisted of 75 men and 15 women, with a mean age of 65.7 years (range, 44–85 years). Fifty-eight patients tested positive for the anti-hepatitis C virus antibody, and 61 patients had previously undergone therapy for HCC. The median AFP and DCP levels were 352.9 ng/mL (range, 0.9–274100 ng/mL) and 1029.0 mAU/mL (range, 6–344740 mAU/mL), respectively. Among 90 patients, 8 (8.9%), 19 (21.1%), and 63 (70.0%) had an elevated level of AFP, elevated level of DCP, or elevated level of both these tumor markers, respectively.

**Table 1:** Patient Characteristics.

	N = 90
Age (Mean ± SD)	65.7 ± 9.0
Sex (M/F)	75/15
Etiology (HCV/others)	58/32
Child-Pugh score (A/B)	44/46
Stage (3/4) ^a^	28/62
Metastases (+/-)	23/67
CR + PR/SD + PD	31/59
Pretreatment (+/-)	61/29
Additional therapy (+/-)	54/36
Overall survival (months)	17.8 ± 23.5
AFP (ng/mL)	352.9 (0.9–274100.0)
DCP (mAU/mL)	1029.0 (6.0–344740.0)
AFP responders/non-responders	27/44
DCP responders/non-responders	39/43

HCV, Hepatitis C virus; AFP, alpha-fetoprotein; DCP, des-gamma-carboxy prothrombin; Vp, portal vein tumor thrombosis; CR, complete response; PR, partial response, SD, stable disease; PD, progressive disease.

^a^According to the Liver Cancer Study Group of Japan

Of the 90 patients, 2 were treated with HAIC using low-dose FP, 62 were treated with HAIC using low-dose FP and isovorin, 15 were treated with combination therapy consisting of low-dose FP, isovorin, and subcutaneous IFN-alpha-2b, and 11 were treated with combination therapy consisting of low-dose FP, isovorin, and subcutaneous PEG-IFN-alpha-2b. The clinical observation period following completion of HAIC treatment ranged from 42–4597 days.

### Response to Therapy and Predictors of Response

The response to therapy is summarized in Table 2. Of the 90 HCC patients, 1 patient (1.1%) exhibited CR, 30 (33.3%) exhibited PR, 39 (43.3%) exhibited SD, and 20 (20.2%) exhibited PD. The response rate, calculated as the number of patients exhibiting CR or PR divided by the total number of patients, was 34.4%.

**Table 2:** Response to therapy.

		CR	PR	SD	PD	Response rate
	Total case (N = 90)	1	30	39	20	34.40%
AFP response	AFP responders (N = 27)	1	13	10	3	51.90%
	AFP non-responders (N = 44)	0	9	20	15	20.50%
	Total (N = 71)	1	22	30	18	32.40%
						* *P* = 0.023
DCP response	DCP responders (N = 39)	1	21	11	6	56.40%
	DCP non-responders (N = 43)	0	6	25	12	14.00%
	Total (N = 82)	1	27	36	18	34.20%
						* *P* < 0.001

AFP, alpha-fetoprotein; DCP, des-gamma-carboxyprothrombin

CR, complete response; PR, partial response; SD, stable disease; PD, progressive disease

*Responders versus non-responders.

With respect to AFP levels, among the 71 patients, the treatment response rate was 51.9% among AFP responders and 20.5% among AFP non-responders (*p* = 0.023). There were no significant differences in clinical characteristics between AFP responders and AFP non-responders (S2 Table). With respect to DCP levels, among the 82 patients, the treatment response rate was 56.4% in DCP responders and 14.0% in DCP non-responders (*p* < 0.001). There were no significant differences in clinical characteristics between DCP responders and DCP non-responders (S3 Table).

Predictors of response to therapy are shown in Table 3. Of the 13 factors analyzed by univariate analysis, two factors were significant predictors for response: AFP response (*p* = 0.006) and DCP response (*p* < 0.001). Multiple logistic regression analysis identified DCP response (odds ratio [OR] 16.03; 95% confidence interval [CI] 3.76–112.53; *p* < 0.001) as an independent predictor of response to therapy.

**Table 3:** Predictors of response to therapy.

Factors	Univariate analysis	Multivariate analysis		
	*P* value	Odds ratio	95% CI	*P* value
Age	0.47			
Sex	0.623			
Etiology	0.345			
Child-Pugh score	0.608			
Stage ^a^	0.263			
Metastases	0.637			
Pretreatment	0.149			
Vp	0.206			
Vv	0.887			
AFP	0.786			
DCP	0.779			
AFP responder	0.006	3.53	0.97–13.80	0.055
DCP responder	< 0.001	16.03	3.76–112.53	<0.001

AFP, alpha-fetoprotein; DCP, des-gamma-carboxyprothrombin; Vp, portal tumor thrombosis; Vv, hepatic vein tumor thrombosis

^a^ According to the Liver Cancer Study Group of Japan

### Patient Survival and Predictors of Survival

**Fig 1:**
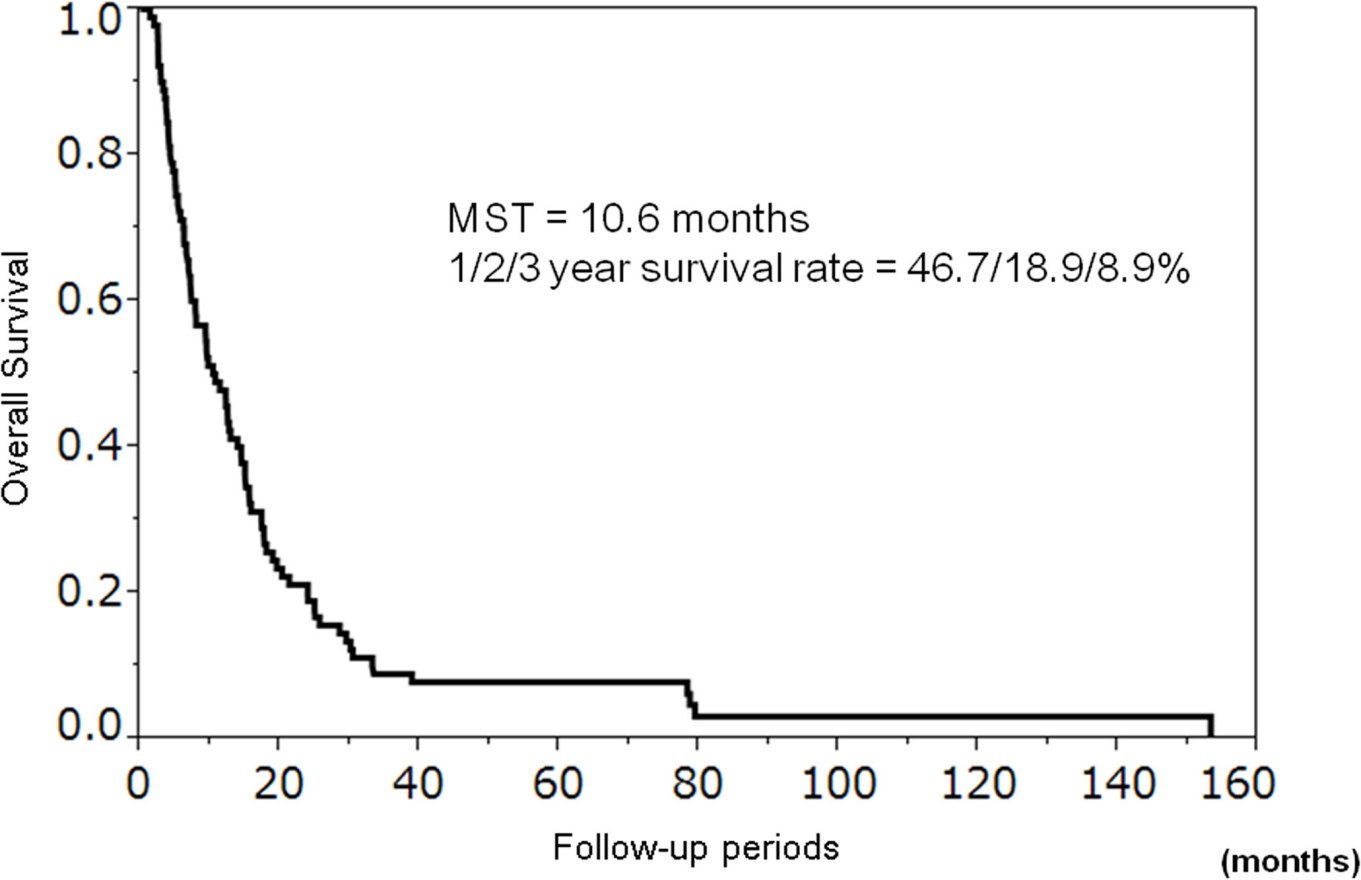
Cumulative survival of 90 advanced hepatocellular carcinoma patients receiving hepatic arterial infusion chemotherapy. The survival rates at 1, 2, and 3 years were 46.7%, 18.9%, and 8.9%, respectively. The median survival time (MST) was 10.6 months.

**Fig 2:**
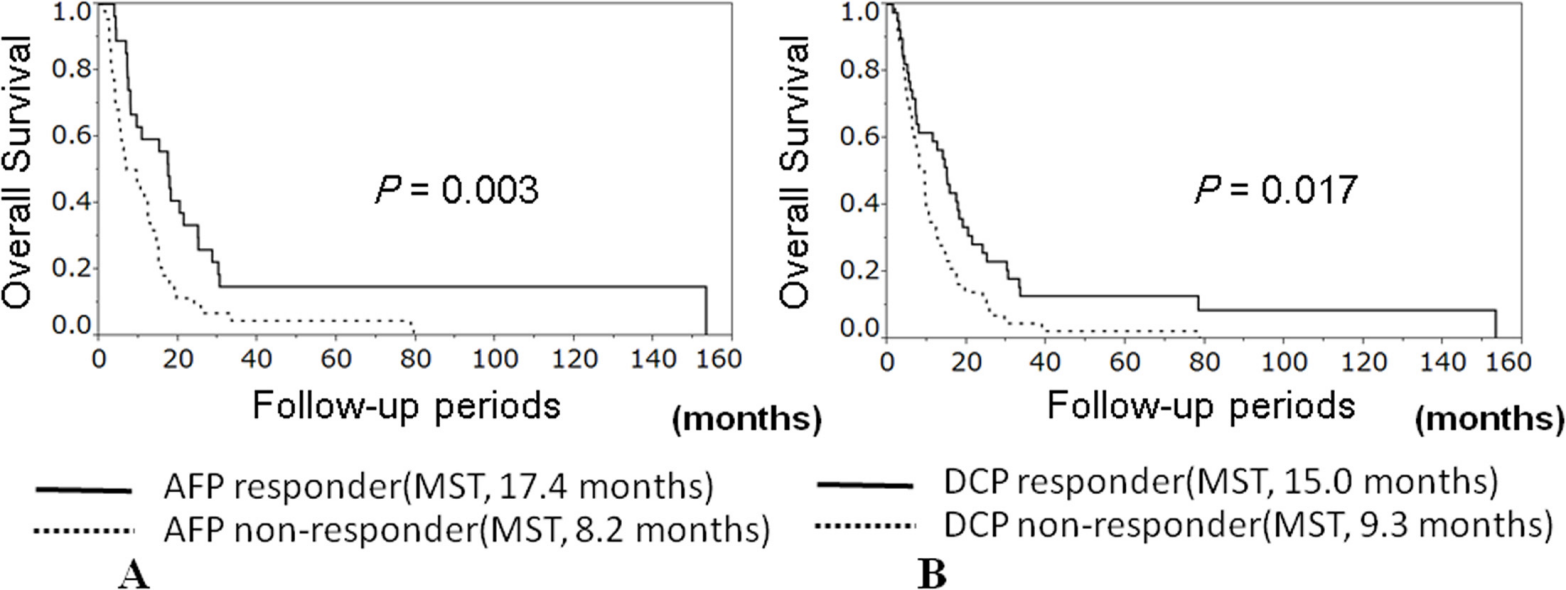
(A) Cumulative survival rates of patients subdivided by alpha-fetoprotein (AFP) response to hepatic arterial infusion chemotherapy. AFP responders (median survival time; MST, 17.4 months) showed significantly longer overall survival than non-responders (MST, 8.2 months; *p* = 0.003). **(B) Cumulative survival rates of patients subdivided by des-gamma-carboxy prothrombin (DCP) response to hepatic arterial infusion chemotherapy.** DCP responders (MST, 15.0 months) showed significantly longer survival than non-responders (MST, 9.3 months; *p* = 0.017).

The overall survival rates at 1, 2, and 3 years were 46.7%, 18.9%, and 8.9%, respectively (Fig 1). The MST was 10.6 months. Predictors of survival are shown in Table 4. Of the 13 factors analyzed using univariate analysis, two factors were significant predictors of survival: AFP response (*p* = 0.003) and DCP response (*p* = 0.017). AFP and DCP responders showed significantly longer overall survival than non-responders (AFP: MST, 17.4 vs. 8.2 months; DCP: MST, 15.0 vs. 9.3 months; Fig 2). In multivariate analysis, Child-Pugh score A (hazard ratio [HR] 1.99; 95% CI 1.13–3.51; *p* = 0.018), AFP response (HR 2.17; 95% CI 1.23–3.92; *p* = 0.007), and DCP response (HR 1.90, 95% CI 1.06–3.42; *p* = 0.030) were independent prognostic predictors (Table 4).

**Table 4:** Predictors of survival after HAIC treatment.

Factors	Univariate analysis			Multivariate analysis		
	Hazard ratio	95%CI	*P* value	Hazard ratio	95% CI	*P* value
Age	1.26	0.82–1.98	0.294			
Sex	1.11	0.65–2.02	0.708			
Etiology	0.73	0.47–1.15	0.176			
Child-Pugh score	1.49	0.97–2.29	0.07	1.99	1.13–3.51	0.018
Stage ^a^	1.25	0.80–2.00	0.337			
Metastases	1.2	0.71–1.94	0.479			
Pretreatment	0.95	0.60–1.56	0.846			
Vp	1.03	0.67–1.59	0.895			
Vv	0.67	0.31–1.24	0.208			
AFP	1.32	0.86–2.08	0.209			
DCP	0.97	0.63–1.50	0.904			
AFP responder	2.13	1.29–3.62	0.003	2.17	1.23–3.92	0.007
DCP responder	1.74	1.10–2.74	0.017	1.9	1.06–3.42	0.03

AFP, alpha-fetoprotein; DCP, des-gamma-carboxyprothrombin; Vp, portal tumor thrombosis; Vv, hepatic vein tumor thrombosis

^a^ According to the Liver Cancer Study Group of Japan

**Fig 3:**
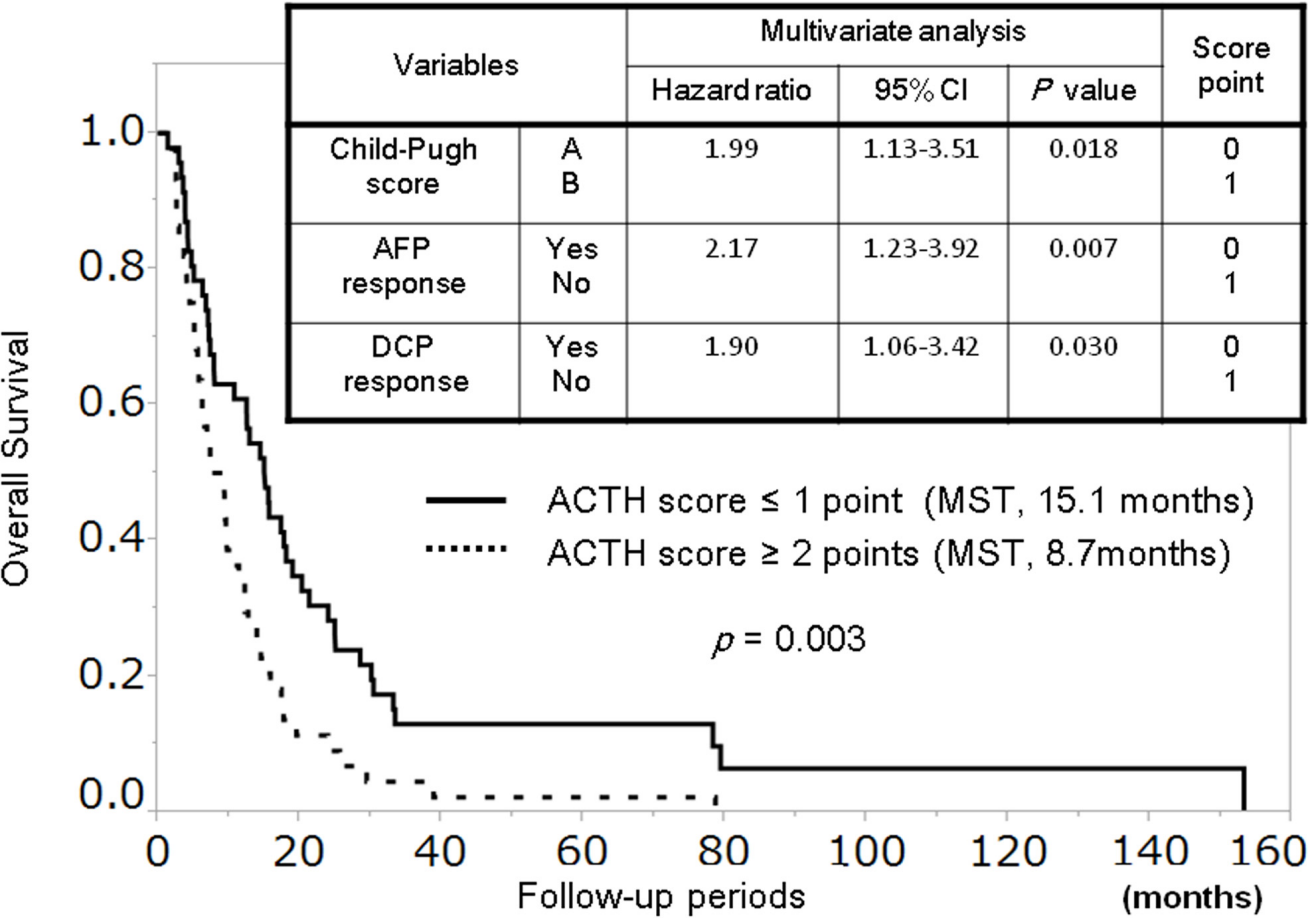
The ACTH score predicts overall survival. Patients stratified into two groups according to their ACTH score showed significantly different prognoses (≤ 1 vs. ≥ 2 points: median survival time, 15.1 vs. 8.7 months; *p* = 0.003).

We developed an Assessment for Continuous Treatment with HAIC (ACTH) score based on the HR of the significant variables obtained from multivariate analysis, ranging from 0 to 3. The ACTH score consists of 3 parameters: Child-Pugh score, AFP response, and DCP response, and is calculated as follows: Child-Pugh score (A = 0, B = 1), AFP response (yes = 0, no = 1), and DCP response (yes = 0, no = 1) (Fig 3). Consequently, a change in the normal range of AFP and DCP between baseline and half a course of HAIC is given a score of 0.

### The ACTH Score Predicts Overall Survival

Patients stratified into two groups according to their ACTH score; 46 (51.1%) and 44 patients (48.9%) were classified as having an ACTH score ≤ 1 and ≥ 2, respectively. These two groups showed significantly different prognoses (≤ 1 vs. ≥ 2 points: MST, 15.1 vs. 8.7 months; *p* = 0.003; Fig 3).

## Discussion

The prognosis of advanced HCC, especially in the presence of vascular invasion and/or extrahepatic spread, remains poor. In patients with advanced HCC who did not receive active therapy, the MST is 6–7 months [28]. In Japan, HAIC and sorafenib are the recommended treatments for advanced HCC [9]. However, there are no established criteria to determine which of these would be the most appropriate. Since 1997, we have been performing HAIC based on low-dose FP in advanced HCC patients [15, 20, 22–24, 29], as it has been shown that HAIC substantially prolongs survival in patients who achieve CR or PR. However, it has been shown that current therapies, worsen the outcome of HAIC refractory patients [15], with the exception of sorafenib, which may improve prognosis [30]. It is therefore important to evaluate the response to HAIC early in the treatment period. Until recently, we have assessed the response to HAIC after one full course (approximately 5 weeks) of treatment. If the response to HAIC can be evaluated during treatment, we can switch HAIC refractory patients to alternate treatments such as sorafenib or new therapy within a clinical trial early in the treatment period. Recently, many staging systems for HCC that can predict patient prognosis, such those developed by the Barcelona Clinic Liver Cancer (BCLC) [31], the Cancer of the Liver Italian Program (CLIP) [32], and the Gropu d’Etude et de Tritement du Carcinoma Hpatocellulaire (GETCH) [33], as well as the Chinese University Prognostic Index (CUPI) [34], and the Japan Integrated Staging (JIS) [19] have been reported. However, these staging systems are not therapeutic assessment scores, and there are currently no therapeutic assessment scores to aid decision-making with regard to continuous HAIC treatment. Therefore, we developed a new therapeutic assessment score for advanced HCC patients receiving HAIC.

AFP and DCP are used in the diagnosis and surveillance of HCC [35, 36]. However, there is yet no consensus as to the usefulness of changes in tumor markers for assessing the response to HCC treatment [37]. It has been reported that changes in AFP levels after systemic therapy [38], locoregional therapy [39], and HAIC [26] may be predictive of treatment outcome. Therefore, we added two factors related to changes between pre-treatment and the end of a half-course of HAIC (AFP and DCP responses) to 11 pre-treatment factors. While a previous study assessed changes in tumor marker levels at the completion of two cycles of HAIC [26], no clinical studies have examined AFP and DCP responses at the mid-cycle of HAIC. We found that there was a significant correlation between the ratio of AFP/DCP after half a course of HAIC and after one complete course of HAIC compared to baseline (AFP: r = 0.583, *p* < 0.001, DCP: r = 0.796, *p* < 0.001; S1 Fig). These findings show that the AFP and DCP levels at the end of half a course of HAIC reflect those after a full course of HAIC.

For predictors of response, the univariate analysis showed that AFP response and DCP response were independent parameters, and the multivariate analysis further identified DCP response (OR, 16.03; 95% CI, 3.76–112.53; *p* < 0.001). The mean half-life of DCP is shorter than that of AFP (DCP, 3.2 days; AFP, 6 days) [40]. Thus, changes in DCP may be more useful than changes in AFP for assessing the early treatment response to HAIC.

We also assessed whether these factors could predict survival. The univariate analysis showed that AFP response and DCP response were independent parameters, and the multivariate analysis further identified Child-Pugh score (HR, 1.99; 95% CI, 1.13–3.51; *p* = 0.018), AFP response (HR, 2.17; 95% CI, 1.23–3.92; *p* = 0.007), and DCP response (HR, 1.90; 95% CI, 1.06–3.42; *p* = 0.030). Although the Child-Pugh score has previously been identified as significant prognostic factor in many reports [5, 13, 21], only a few of these reports related to changes in tumor marker levels [26, 41].

Therefore, we developed the ACTH score based on the HR of the significant variables identified in multivariate analysis. The ACTH score consists of 3 parameters: Child-Pugh score, AFP response, and DCP response. AFP and DCP have been used as complementary tumor markers [35, 42]. Elevated levels of AFP or DCP were associated with malignant potential such as vascular invasion, tumor differentiation, and intrahepatic metastases, but they have different relationships with a number of clinicopathological variables of HCC [42]. Although HCC patients with either elevated AFP or elevated DCP before HAIC were included in this study, we defined a non-response with respect to these markers both as a reduction of < 20% from baseline and an increase from baseline after half a course of HAIC. In addition, a change in the normal range of AFP and DCP between baseline and half a course of HAIC was defined as score 0. Toyoda, et al. reported that the number of elevated post-treatment tumor markers was significantly associated with survival in HCC patients [43], supporting this approach. The ACTH score identified two distinct groups with different prognoses (ACTH score ≤ 1 vs. ≥ 2 points: MST, 15.1 vs. 8.7 months; *p* = 0.003; Fig 3). When we analyzed 63 patients with elevated levels of both AFP and DCP, and stratified them into two groups according to this score, there was a significantly different prognosis between the groups (ACTH score ≤ 1 vs. ≥ 2 points: MST, 17.3 vs. 6.7 months; *p* = 0.001) (S2 Fig).

**Fig 4:**
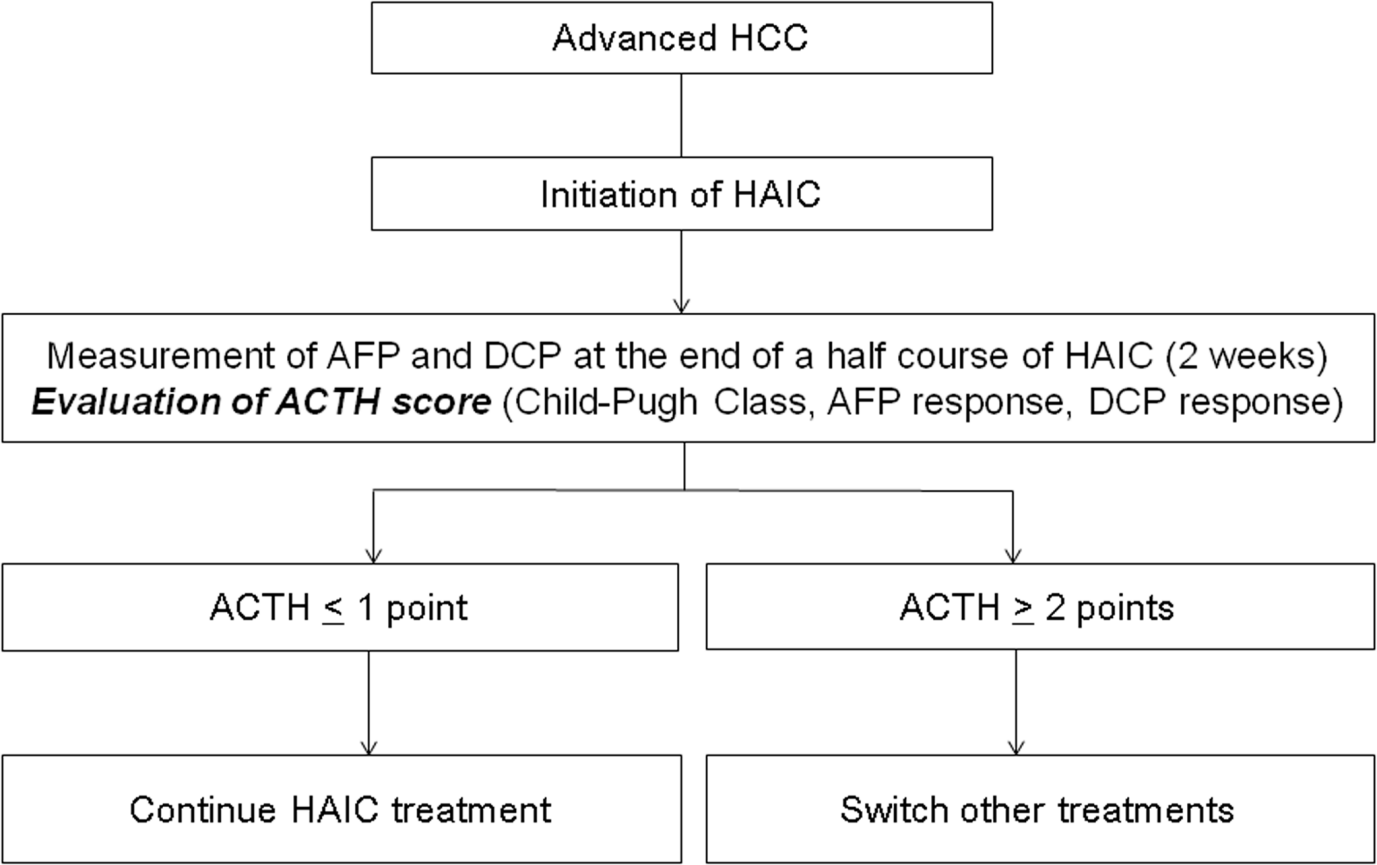
The treatment strategy for advanced hepatocellular carcinoma according to the ACTH score. AFP and DCP were measured after half a course of HAIC (2 weeks after the initiation of HAIC), and the ACTH score was evaluated. For patients with a score ≤ 1, HAIC treatment would be continued, and for patients with a score ≥ 2, a second line therapy such as sorafenib and/or a new clinical trial would be a better option.

For patients with a score ≤ 1, HAIC treatment would be continued, and for patients with a score ≥ 2, a second line therapy such as sorafenib and/or participation in a new clinical trial would be a better option (Fig 4).

There are limitations to this study. First, the chemotherapeutic regimens used were not uniform between all patients, even though all the regimens were based on low-dose FP. However, it was previously shown that there are no significant differences in response or survival between these regimens [29]. A validation study is required in HCC patients treated with a uniform low-dose FP regimen or other HAIC regimens. Second, this was a retrospective cohort study examining a small population. However, there have only been a few studies of HAIC in more than 100 advanced HCC patients [11–13, 29, 44, 45]. A prospective study of a larger patient population is necessary. Third, this score cannot be used in patients with normal levels of tumor markers. However, patients with high tumor marker levels tend to have advanced HCC, especially if there is also vascular invasion and/or extrahepatic spread [46]; indeed, 90 of the 130 patients (69%) could be enrolled in this study.

In conclusion, the ACTH score, which consists of 3 simple factors, may help in the therapeutic assessment of HCC patients receiving HAIC, and could make it possible to use a new treatment earlier for non-responders. This may in turn significantly improve patient survival.

## Supporting Information

S1 Fig Correlation between the AFP:DCP ratio at base line and after half a course of HAIC. There was a significant correlation between the AFP:DCP ratio at baseline and after half a course of HAIC. (AFP: r = 0.583; *p* < 0.001, DCP: r = 0.796; *p* < 0.001).
(TIF)

S2 Fig The ACTH score predicts overall survival in advanced hepatocellular carcinoma patients with AFP and DCP. When we analyzed 63 patients with elevated levels of both AFP and DCP, and stratified into two groups according to this score, there was a significantly different prognosis between the groups (ACTH score ≤ 1 vs. ≥ 2 points: MST, 17.3 vs. 6.7 months; *p* = 0.001).
(TIF)

S1 Table Patient Characteristics. (TIF)

S2 Table Characteristics of patients with elevated AFP at baseline. (TIF)

S3 Table Characteristics of patients with elevated DCP at baseline. (TIF)
