# Supplementary figures and images for: A New Therapeutic Assessment Score for Advanced Hepatocellular Carcinoma Patients Receiving Hepatic Arterial Infusion Chemotherapy
Source: PLoS One. 2015 May 20;10(5):e0126649. doi: 10.1371/journal.pone.0126649 (PMC4439162; doi:10.1371/journal.pone.0126649)

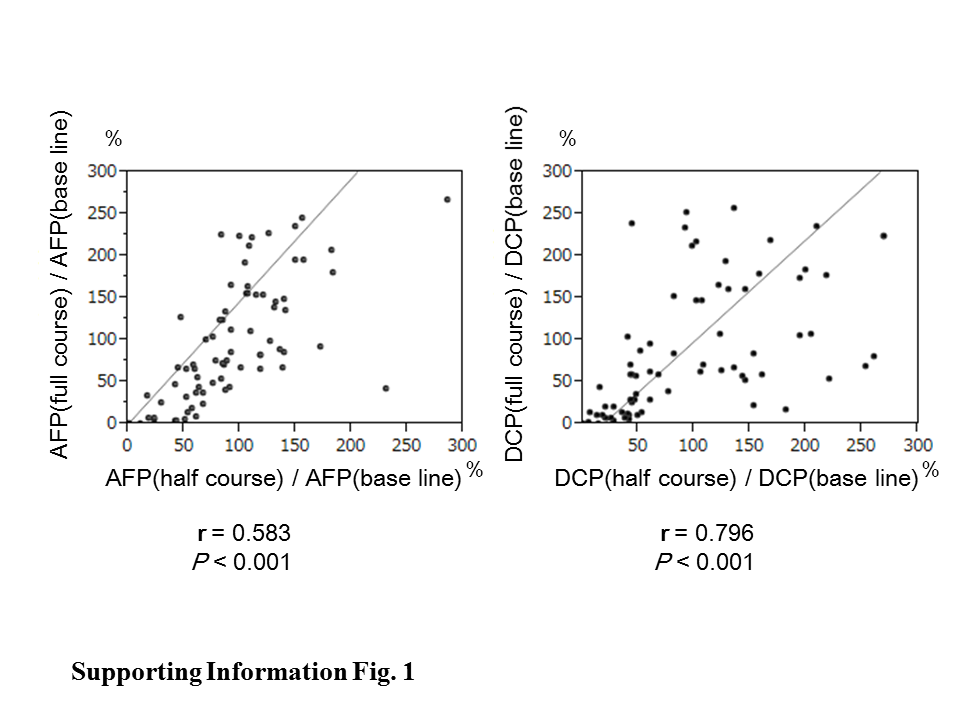

Supplement: S1 Fig — There was a significant correlation between the AFP:DCP ratio at baseline and after half a course of HAIC. (AFP: r = 0.583; p < 0.001, DCP: r = 0.796; p < 0.001). (TIF) [file pone.0126649.s001.tif]

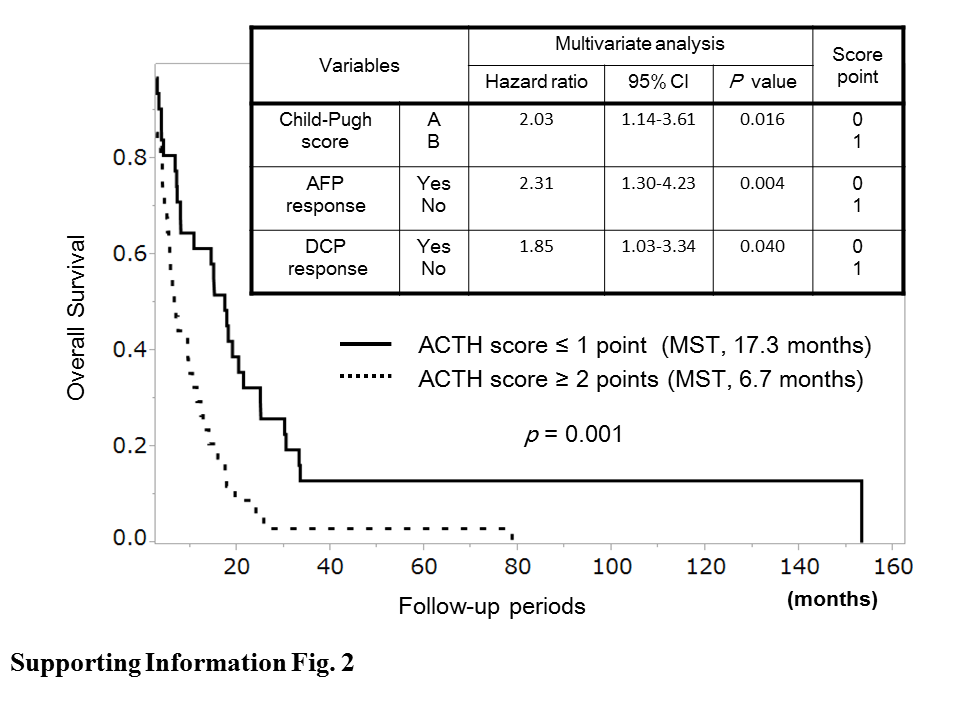

Supplement: S2 Fig — When we analyzed 63 patients with elevated levels of both AFP and DCP, and stratified into two groups according to this score, there was a significantly different prognosis between the groups (ACTH score ≤ 1 vs. ≥ 2 points: MST, 17.3 vs. 6.7 months; p = 0.001). (TIF) [file pone.0126649.s002.tif]

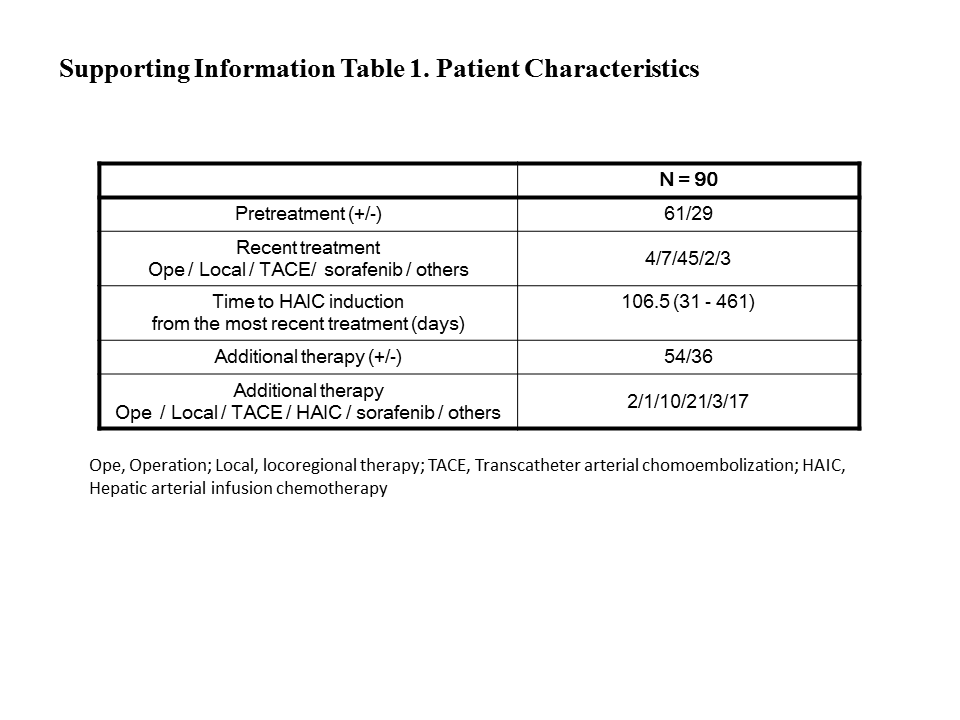

Supplement: S1 Table — (TIF) [file pone.0126649.s003.tif]

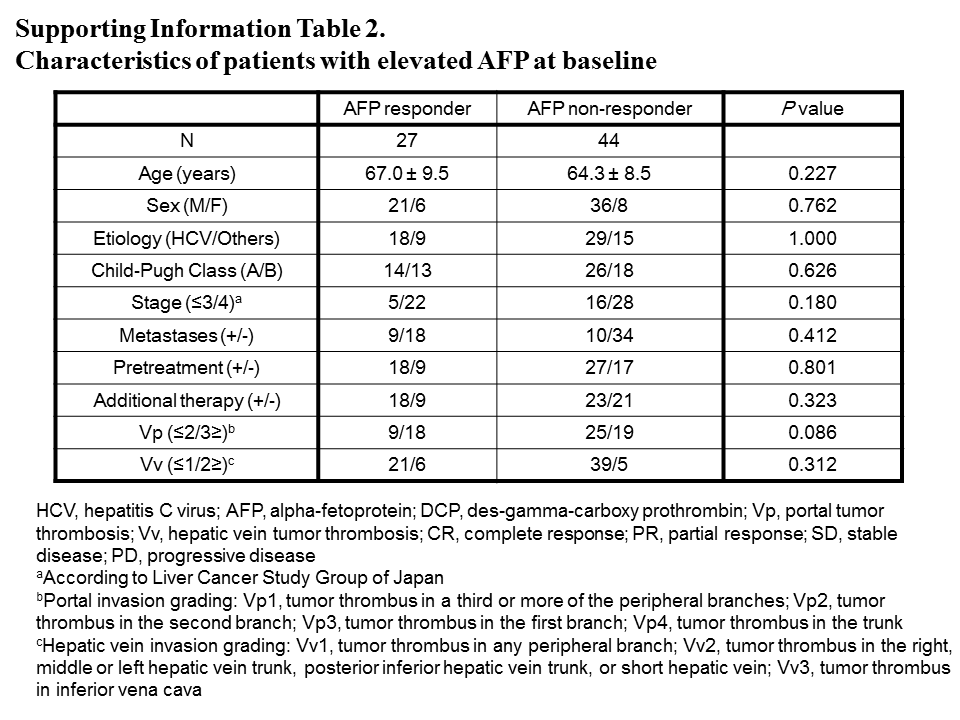

Supplement: S2 Table — (TIF) [file pone.0126649.s004.tif]

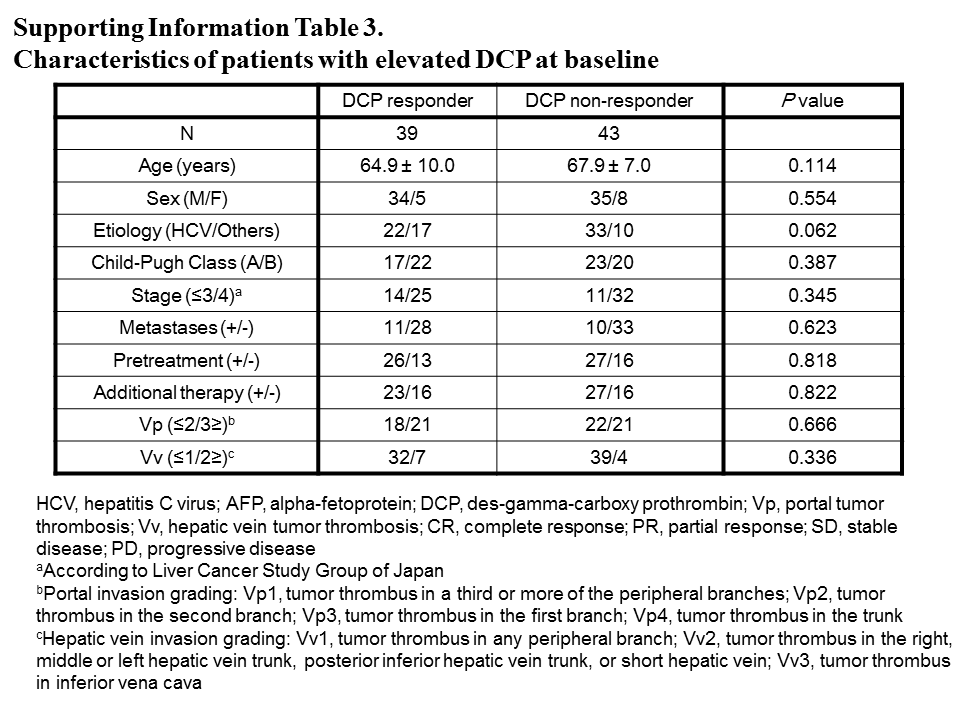

Supplement: S3 Table — (TIF) [file pone.0126649.s005.tif]
